# Immediate versus conventional loading implants with fixed prosthesis - A clinical and radiological study

**DOI:** 10.6026/973206300220405

**Published:** 2026-01-31

**Authors:** Sahba Hassan, Dibya Kumari, Shailesh Jain, Nishant Gupta, Lalit Kumar, Surabhi Duggal

**Affiliations:** 1 Department of Prosthodontics and Crown & Bridge, ITS Dental College, Greater Noida, Uttar Pradesh, India; 2 Department of Conservative dentistry and Endodontics, Narisnbhai Patel Dental College and Hospital, Sankalchand Patel University, Visnagar, North Gujarat, India; 3 Department of Prosthodontics and Crown & Bridge, Bhojia Dental College, Baddi, Himachal Pradesh, India; 4 Department of Orthodontics and Dentofacial Orthopedics, Santosh Dental College and Hospital, Santosh Deemed to be University, Uttar Pradesh, India; 5 Department of Prosthodontics, Medical Officer (Dental) CHC Chandi, Solan, Himachal Pradesh, India; 6 Department of Prosthodontics and Crown & Bridge, School of Dental Sciences, Sharda university, Greater Noida, Uttar Pradesh, India

**Keywords:** Immediate loading, conventional loading, dental implant, marginal bone loss, fixed prosthesis

## Abstract

The success of dental implants is influenced by the timing of prosthetic loading. Therefore, it is of interest to compare the clinical and radiological outcomes of immediate loading (IL) versus conventional loading (CL) of dental implants supporting fixed prostheses. Hence, a total of 30 patients were randomly assigned to either IL or CL groups. Clinical parameters such as implant stability (measured by Periotest), peri-implant soft tissue health and radiographic marginal bone loss (MBL) were evaluated over a 12-month period. Results indicated that both groups demonstrated high implant survival rates (96.6% IL vs 100% CL), with no statistically significant differences in MBL at 3, 6 and 12 months (p > 0.05). However, IL provided faster functional rehabilitation and patient satisfaction. Thus, we show the clinical viability of immediate loading protocols in appropriately selected cases, without compromising implant stability or bone levels.

## Background:

Dental implants are a widely accepted treatment modality for replacing missing teeth due to their high success rates and long-term predictability [1]. Traditionally, implants were left unloaded for 3-6 months to allow osseointegration before prosthetic placement-a protocol known as conventional loading (CL). However, this approach prolongs treatment time and patient discomfort [2]. Immediate loading (IL), defined as loading the implant within 48 hours of placement, has emerged as a promising alternative. IL shortens the rehabilitation period and provides immediate function and esthetics [3, 4]. The concept is supported by studies suggesting that primary stability and absence of micromotion are critical for successful osseointegration even under functional load [5]. Several randomized controlled trials and systematic reviews have compared IL and CL protocols, often showing comparable implant survival and bone loss outcomes [6, 7- 8]. However, concerns remain regarding the long-term success of IL, particularly in cases with compromised bone quality or suboptimal surgical placement [9]. Radiological assessment, especially measurement of marginal bone loss (MBL), is crucial in evaluating implant performance over time [10]. Therefore, it is of interest to compare IL and CL protocols in terms of clinical and radiological outcomes using fixed implant-supported prostheses over a 1-year period.

## Methodology:

This prospective, randomized clinical study was conducted at a tertiary dental care institute to evaluate and compare the clinical and radiological outcomes of immediate versus conventional loading of dental implants with fixed prostheses. A total of 30 patients, aged between 25 and 60 years, with partially edentulous arches requiring implant-supported prostheses in the posterior maxilla or mandible, were selected based on strict inclusion and exclusion criteria. Inclusion criteria included adequate bone volume as verified by cone-beam computed tomography (CBCT), good oral hygiene and the ability to achieve a minimum insertion torque of 35 Ncm during implant placement. Patients with systemic conditions contraindicating implant surgery (such as uncontrolled diabetes), smokers consuming more than 10 cigarettes per day, or those with active periodontal disease were excluded from the study. After obtaining ethical clearance and informed written consent from all participants, the patients were randomly divided into two equal groups: Group A (Immediate Loading, n=15) and Group B (Conventional Loading, n=15). All patients received titanium, screw-type implants with diameters ranging from 3.5 to 4.5 mm and lengths between 10 to 12 mm. Implants were placed using a standard surgical protocol. In Group A, definitive prostheses were delivered within 48 hours post-implant placement. In contrast, Group B underwent a conventional loading protocol, with prosthesis placement after a healing period of 3 months to allow for osseointegration. Clinical evaluations were performed at baseline and subsequently at 3, 6 and 12 months. Parameters assessed included implant stability (measured using a Periotest device), plaque index and bleeding on probing. Standardized intraoral periapical radiographs were taken using a paralleling technique to assess marginal bone levels. Radiographic analysis was performed digitally to determine mesial and distal bone loss around each implant. Statistical analysis was carried out using SPSS version 25.0 and comparisons between groups were made using Student's t-test and Chi-square test, with a p-value < 0.05 considered statistically significant.

## Results:

**Figure 1:**
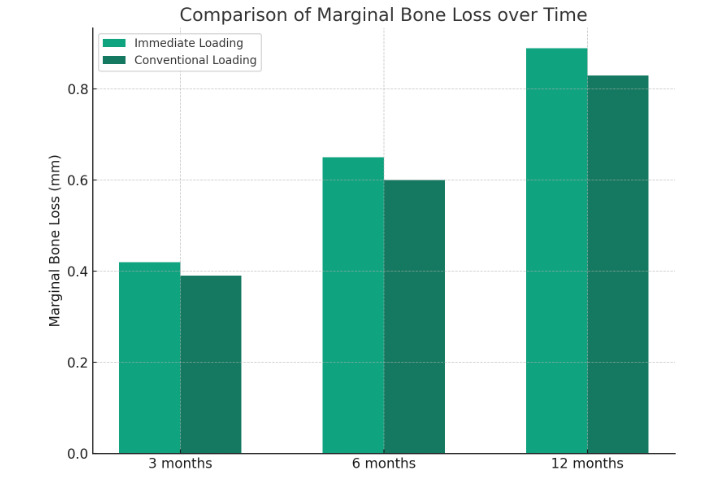
Comparison of marginal bone loss over time

Out of 30 patients included in the study, 15 were treated under the Immediate Loading (IL) protocol and 15 under the Conventional Loading (CL) protocol. The overall implant survival rate was 98.3%. One implant failure occurred in the IL group within the first month due to poor primary stability, resulting in a survival rate of 96.6% for IL compared to 100% in the CL group; however, this difference was not statistically significant (p > 0.05). Implant stability, assessed using the Periotest device and improved consistently in both groups over the study period. At baseline, the mean Periotest value (PTV) in the IL group was -1.5 ± 1.0, is improving to -3.2 ± 0.8 at 12 months. In the CL group, the baseline PTV was -2.0 ± 0.9, reaching -3.4 ± 0.7 at 12 months. The differences between the groups at each time point were not statistically significant (p > 0.05) (Table 1). Marginal bone loss (MBL) was measured radiographically at mesial and distal aspects of each implant. At 3 months, mean MBL was 0.42 ± 0.13 mm in the IL group and 0.39 ± 0.10 mm in the CL group. At 6 months, the values were 0.65 ± 0.20 mm and 0.60 ± 0.18 mm, respectively. By 12 months, MBL reached 0.89 ± 0.25 mm for IL and 0.83 ± 0.22 mm for CL. Though the IL group showed slightly higher bone loss throughout, the differences were statistically insignificant (p > 0.05) (Figure 1). No significant differences were observed between the groups in terms of plaque index or bleeding on probing at any follow-up visit, indicating similar peri-implant soft tissue health.

## Discussion:

The current study evaluated the clinical and radiological outcomes of immediate versus conventional loading of dental implants supporting fixed prostheses over 12 months. Implant survival rates were high in both groups, with one implant failure in the immediate loading group (96.6%) and no failures in the conventional group (100%). This is consistent with previous reports showing comparable success between the two protocols [6, 11]. Implant stability, measured by Periotest, improved over time in both groups, with no significant difference at any time point. This supports the notion that initial primary stability and proper case selection are more critical than loading timing in determining implant success [5, 12]. Marginal bone loss (MBL) is a critical indicator of peri-implant health. In our study, mean MBL was slightly higher in the IL group at all time points but did not reach statistical significance. These findings align with systematic reviews which have shown minor, non-significant increases in bone loss associated with IL [7, 13]. Patient satisfaction, although not quantified here, was subjectively reported to be higher in the IL group due to faster restoration of function and esthetics. This has been a consistent finding in previous literature [14]. However, clinicians must carefully select candidates for IL, as it requires good bone quality, high insertion torque and rigid prosthetic splinting to mitigate micromotion [15]. Concerns have been raised about IL increasing the risk of peri-implantitis due to bacterial colonization during the healing phase [16] Chen *et al.* (2019) demonstrated through a comprehensive meta-analysis that IL yields implant survival and marginal bone level outcomes comparable to conventional loading when adequate primary stability is achieved. This aligns with the current results, where implant survival exceeded 96% in both groups [17]. Similarly, Hadilou *et al.* (2021) reported that even short implants, when immediately loaded under controlled occlusal conditions, show predictable performance with no significant increase in failure risk- further supporting the viability of IL in appropriately selected cases [18]. Long-term radiological outcomes also support our findings. Wang *et al.* (2022) observed stable marginal bone levels over a 6.5-year follow-up with immediately loaded self-tapping implants, indicating that early bone remodeling does not differ significantly from conventional loading once osseointegration is established [19]. This mirrors the stable bone loss pattern seen in the present study during the first year. More recently, Spinato *et al.* (2025) found minimal marginal bone changes after prosthesis delivery in tissue-level implants, reinforcing that prosthetic loading timing has limited influence on peri-implant bone stability when biological and mechanical prerequisites are met [20]. Although short-term outcomes are promising, longer-term data are necessary to confirm the predictability of IL beyond 1 year. This study has limitations, including a relatively small sample size and short follow-up period. Future multicenter trials with larger cohorts and patient-reported outcome measures (PROMs) could provide more robust evidence.

## Conclusion:

Immediate loading of dental implants supporting fixed prostheses demonstrates comparable clinical and radiological outcomes to conventional loading in well-selected cases. While immediate loading offers advantages of reduced treatment time and improved patient satisfaction, careful patient selection and strict clinical protocols are essential to ensure long-term success. Both protocols remain viable, with clinical judgment playing a key role in treatment planning.

## Figures and Tables

**Table 1:** Comparison of clinical and radiographic outcomes between immediate and conventional loading groups

**Parameter**	**Time Point**	**Immediate Loading (Mean ± SD)**	**Conventional Loading (Mean ± SD)**	**p-value**
Implant Survival Rate (%)	12 months	96.60%	100%	>0.05
Periotest Value (PTV)	Baseline	-1.5 ± 1.0	-2.0 ± 0.9	>0.05
	12 months	-3.2 ± 0.8	-3.4 ± 0.7	>0.05
Marginal Bone Loss (mm)	3 months	0.42 ± 0.13	0.39 ± 0.10	>0.05
	6 months	0.65 ± 0.20	0.60 ± 0.18	>0.05
	12 months	0.89 ± 0.25	0.83 ± 0.22	>0.05
